# The relationship between urban environment and the inflammatory bowel diseases: a systematic review and meta-analysis

**DOI:** 10.1186/1471-230X-12-51

**Published:** 2012-05-24

**Authors:** Ing Shian Soon, Natalie A Molodecky, Doreen M Rabi, William A Ghali, Herman W Barkema, Gilaad G Kaplan

**Affiliations:** 1 Departments of Medicine, University of Calgary, Alberta, Canada; 2 Department of Community Health Sciences, University of Calgary, Alberta, Canada; 3 Production Animal Health, University of Calgary, Alberta, Canada; 4 Departments of Medicine and Community Health Sciences, University of Calgary, Teaching Research and Wellness Center, 3280 Hospital Drive NW, 6th Floor, Room 6D17, Calgary, AB, T2N 4 N1, Alberta, Canada

**Keywords:** Inflammatory bowel disease, Urban population, Risk factors

## Abstract

**Background:**

The objective of this study was to conduct a systematic review with meta-analysis of studies assessing the association between living in an urban environment and the development of the Crohn’s disease (CD) or ulcerative colitis (UC).

**Methods:**

A systematic literature search of MEDLINE (1950-Oct. 2009) and EMBASE (1980-Oct. 2009) was conducted to identify studies investigating the relationship between urban environment and IBD. Cohort and case–control studies were analyzed using incidence rate ratio (IRR) or odds ratio (OR) with 95 % confidence intervals (CIs), respectively. Stratified and sensitivity analyses were performed to explore heterogeneity between studies and assess effects of study quality.

**Results:**

The search strategy retrieved 6940 unique citations and 40 studies were selected for inclusion. Of these, 25 investigated the relationship between urban environment and UC and 30 investigated this relationship with CD. Included in our analysis were 7 case–control UC studies, 9 case–control CD studies, 18 cohort UC studies and 21 cohort CD studies. Based on a random effects model, the pooled IRRs for urban compared to rural environment for UC and CD studies were 1.17 (1.03, 1.32) and 1.42 (1.26, 1.60), respectively. These associations persisted across multiple stratified and sensitivity analyses exploring clinical and study quality factors. Heterogeneity was observed in the cohort studies for both UC and CD, whereas statistically significant heterogeneity was not observed for the case–control studies.

**Conclusions:**

A positive association between urban environment and both CD and UC was found. Heterogeneity may be explained by differences in study design and quality factors.

## Background

The etiology of the inflammatory bowel diseases (IBD) has been extensively studied [1], however, the environmental determinants of disease pathogenesis are not fully understood [2,3]. IBD is widely believed to be associated with industrialization of nations. This hypothesis is supported by the significant geographic variation in IBD with the highest incidence rate of IBD in North America and Europe [4]. Migrant studies have demonstrated that immigrants, and particularly their offspring, from low prevalent regions acquire a similar risk of IBD as the local population [5]. Furthermore, the incidence of IBD is steadily rising in several developing nations as they have become industrialized [6,7]. Within a country the incidence of IBD has been proposed to be higher in urban versus rural areas [8,9].

Although numerous studies have investigated the association between urban environment and IBD, findings remain inconsistent. An urban association has been considered as far back as 1963, when Acheson and Nefzger suggested a positive relationship between urban areas and ulcerative colitis (UC) [10]. Many observational studies have subsequently shown an increase in UC and Crohn's disease (CD) incidence in more densely populated areas [8,11-15]. Numerous studies, however, have failed to find an association between urban exposure and IBD [4,16], while others have shown an inverse association [17]. Establishing whether the risk of IBD is greater in urban environments is important because environmental exposures in urban societies are significantly different than those in rural areas. This information may direct research initiatives on specific environmental risk factors of IBD and establish distribution of IBD burden in society.

A systematic analysis of the association between living in an urban environment and IBD has yet to be conducted. Thus, we performed a systematic review and meta-analysis of all case–control and cohort studies that explored the association between residing in an urban region and IBD in order to determine whether the risk of CD and/or UC was increased in urban as compared to rural areas.

## Methods

### Search strategy

We conducted a systematic literature search using a predetermined protocol and in accordance with the quality of reporting meta-analyses of observational studies (MOOSE) [18]. We searched two computer-stored databases, Medline (1950-present) and Embase (Excerpta Medica Database; 1980-present) for studies describing the association between urban environment and IBD as of October 2009. The search strategy for Medline and Embase was conducted based on three themes. The first theme, the outcome measure, combined the exploded version of Medical Subject Headings (MESH) “inflammatory bowel disease” or “Colitis, Ulcerative” or “Crohn Disease”. The second theme, the exposure, combined the exploded version of MESH headings “urban population” or “urban health” or “rural population” or “rural health” or “geography”. The third theme combined exploded versions of MESH headings “risk factors” or “risk assessment” or “epidemiology” or “demography”. All the keywords were used to search the titles and abstracts. The search was not limited by language or human subjects to ensure capture of all appropriate papers. Abstracts from the American College of Gastroenterology for 2006, 2007, and 2008, and the American Gastroenterological Association for 2006 and 2007 were reviewed. The reference lists of relevant articles were also reviewed.

### Selection criteria

Two reviewers (N.M. and I.S.) identified articles eligible for further review by performing an initial screen of identified abstracts and titles. Articles were eliminated in this initial screen if they were not observational or did not either investigate environmental risk factors for IBD in a case–control study or investigate epidemiology of IBD using incident cases. Studies that did not report original data and duplicated publications were also excluded. Full-text of the remaining articles were retrieved and systematically reviewed. Articles were considered for inclusion in the second screening if they reported a measure of association between urban environment and UC and/or CD. In both study designs, UC and CD were required to be reported separately for inclusion into the systematic review. Studies that did not report adequate information to calculate incidence rate ratio (IRR) or odds ratio (OR) with 95% confidence interval (CI) were excluded. Disagreement between reviewers was resolved by consensus with third party experts (DR and GK).

### Data extraction

The outcome variable of interest was defined as the presence of UC and/or CD. The exposure variable of interest was residing in an urban versus rural environment. Urban and rural populations were not consistently defined across all studies. Several studies (n = 10 for UC; n = 11 for CD) did not define an urban and/or rural environment [12,13,16,17,19-31]. The studies that defined this exposure used definitions that varied and some papers stratified by multiple levels of exposure (e.g. urban, semi-urban, and rural) [24,32-36]. A priori we identified studies that defined urban as a population greater than 10,000 [37,38]. Secondary variables extracted from the manuscripts included: study design (i.e. case–control or cohort); country of origin; publication year; timing of exposure; source of controls for case–control studies; and information on key indicators of study quality, using MOOSE [18].

We extracted reported OR and IRR with 95% (CIs) or data enabling the calculation of these association measures. Both adjusted and unadjusted values were extracted; though, when available, the adjusted estimates were used.

### Statistical analysis

Meta-analyses were initially conducted by combining cohort and case–control studies using IRRs, defined as the incidence rate of IBD in urban versus rural populations. Given the low prevalence of IBD, the OR would approximate the IRR under the rare disease assumption. Case–control and cohort studies were then analyzed separately using different measures of association. The IRR was used as measure of association for cohort studies and OR was used for case–control studies. The Test of Heterogeneity was performed using a Q statistic (5% level) and random effects models were used because of the presence of heterogeneity between studies. Stratified analyses were performed to explore factors that may explain heterogeneity between studies. Publication year was a priori stratified into three year categories: 1962–1988; 1989–1998; and 1999–2009. Similarly, stratified analyses and meta-regression were performed based on study design (i.e. case–control or cohort study), region of publication, timing of exposure, and source of controls in case–control studies. Sensitivity analyses were performed to exclude studies that did not include a definition of urban/rural region. Further sensitivity analysis was conducted on studies that used >10,000 people as a definition of urban environment [37,38]. Papers that stratified by multiple levels of exposure (e.g. urban, semi-urban, and rural) were analyzed as urban versus rural. The possibility of publication bias was assessed using the Begg tests.

## Results

### Literature search

**Figure 1:**
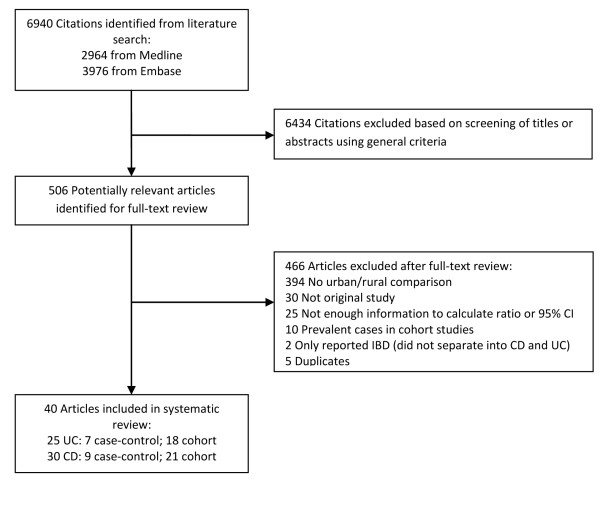
Literature search results.

The search strategy retrieved 6940 unique citations: 2964 from Medline and 3976 from Embase. Of these, 6434 citations were excluded after the first screening based on titles and abstracts, leaving 506 articles for full-text review (Figure 1). The observed agreement between reviewers for eligibility of articles was 97%, corresponding to a kappa statistic of 0.77. Upon full text review of 506 articles, 466 were excluded (see Figure 1 for rationale of exclusions), leaving 40 studies for final inclusion in the systematic review and meta-analysis. Of these 40 studies, 25 investigated the relationship between urban environment and UC and 30 investigated this relationship with CD. Included in our analysis were 7 case–control UC studies, 9 case–control CD studies, 18 cohort UC studies, and 21 cohort CD studies.

### Demographic and study quality characteristics

Characteristics of the 18 cohort studies for UC are shown in Table 1[19-21,23,24,32-35,39-45]. The reported incidence rate ranged from 0.97 to 15.6 per 100,000/year. A definition for urban and/or rural environment was provided for 13 cohort studies. Characteristics of the 7 selected case–control studies for UC are shown in Table 2[8,12,17,26-28,46]. Two of the studies had definitions for urban and/or rural environment. Characteristics of the 21 selected cohort studies for CD are shown in Tables 1[14,15,22,23,25,30,31,36,39-43,45,47-52]. The reported incidence rate of CD ranged from 0.51 to 15.6 per 100,000/year. A definition for urban environment was reported in 16 studies. Characteristics of the nine selected case–control studies for CD are shown in Table 2[8,13,16,17,27,29,46,53]. The studies were based in North America, Australia, Asia and Europe. The association between urban environment and IBD was not the primary outcome of interest in any of the cohort or case–control studies.

**Table 1:** Characteristics of cohort studies for ulcerative colitis and Crohn’s disease studies

**Study characteristics and demographics**					**Study Quality Characteristics**					
**Author (year)**	**Country**	**Study period**	**Number of cases**	**Incidence of UC (per 100,000)**	**Methods of diagnosis defined**	**Diagnosis based on recognized criteria**	**Urban/rural defined**	**Urban population >10,000**	**Time of exposure**	**Multiple levels of exposure**
*Ulcerative Colitis*										
Mate-Jimerez 1994 [39]	Spain	1981-88	111	3.16	Yes	Yes	Yes	Yes	At diagnosis	No
Ekbom 1991 [40]	Sweden	1965-83	2509	10.5	Yes	Yes	Yes	No	At diagnosis	No
Linden 1971 [19]	Finland	1967	223	4.76	No	N/A	No	No	At diagnosis	No
Probert 1992 [32]	UK	1972-80	192	5.33	Yes	Yes	Yes	No	At diagnosis	Yes
Probert 1992 [32]	UK	1981-89	211	7.29	Yes	Yes	Yes	No	At diagnosis	Yes
Niv 1990 [33]	Israel	1967-86	43	2.33	Yes	No	Yes	No	At diagnosis	Yes
Kildebo 1990 [22]	Norway	1983-86	179	13.2	Yes	Yes	No	No	At diagnosis	No
Gheorghe 2004 [23]	Romania	2002-03	163	0.97	Yes	Yes	No	No	At diagnosis	No
Sincic 2006 [41]	Croatia	2000-04	70	4.6	Yes	Yes	Yes	No	At diagnosis	No
Tsianos 1994 [24]	Greece	1982-91	61	4.18	Yes	Yes	No	No	At diagnosis	Yes
Ladas 2005 [34]	Greece	1990-94	56	10.6	Yes	Yes	Yes	Yes	At diagnosis	Yes
Moller 1971 [20]	Finland	1956-67	505	1.07	Yes	Yes	No	No	At diagnosis	No
Blanchard 2001 [42]	Canada	1987-96	1763	15.6	Yes	Yes	Yes	No	At diagnosis	No
Green 2006 [15]	Canada	1990-01	N/A	13.5	No	N/A	Yes	No	At diagnosis	No
Lakatos 2003 [43]	Hungary	1977-01	560	5.89	Yes	Yes	Yes	No	At diagnosis	No
Manousos 1996 [44]	Greece	1990-94	117	8.9	Yes	Yes	Yes	No	At diagnosis	No
Wigley 1962 [35]	New Zealand	N/A	132	N/A	Yes	No	Yes	Yes	At diagnosis	Yes
Latour 1996 [45]	Belgium	1993-94	36	3.5	Yes	Yes	Yes	No	At diagnosis	No
*Crohn’s Disease*										
Kyle 1971 [47]	UK	1955-69	166	1.98	Yes	Yes	Yes	No	At diagnosis	No
Jayanthi 1992 [48]	UK	1972-80	161	2.89	Yes	Yes	Yes	Yes	At diagnosis	No
Jayanthi 1992 [48]	UK	1981-89	233	4.48	Yes	Yes	Yes	Yes	At diagnosis	No
Mate-Jimerez 1994 [39]	Spain	1981-88	57	1.61	Yes	Yes	Yes	Yes	At diagnosis	No
Ekbom 1991 [40]	Sweden	1965-83	1469	6.09	Yes	Yes	Yes	No	At diagnosis	No
Shivananda 1987 [36]	Netherlands	1979-83	54	3.90	Yes	Yes	Yes	No	At diagnosis	Yes
Kildebo 1989 [22]	Norway	1983-86	82	5.85	Yes	Yes	No	No	At diagnosis	No
Kyle 1992 [49]	Scotland	1955-88	856	5.65	Yes	Yes	Yes	No	At diagnosis	No
Manousos 1996 [44]	Greece	1990-94	37	3.00	Yes	Yes	Yes	Yes	At diagnosis	No
Gheorghe 2004 [23]	Romania	2002-03	85	0.51	Yes	Yes	No	No	At diagnosis	No
Sincic 2006 [41]	Croatia	2000-04	100	6.50	Yes	Yes	Yes	No	At diagnosis	No
Phavichitr 2003 [25]	Australia	1971-01	351	1.02	Yes	Yes	No	No	At diagnosis	No
Moum 1996 [50]	Norway	1990-93	225	5.86	Yes	Yes	Yes	No	At diagnosis	No
Blanchard 2001 [42]	Canada	1987-96	1765	15.6	Yes	Yes	Yes	No	At diagnosis	No
Green 2006 [15]	Canada	1990-01	N/A	14.8	No	N/A	Yes	No	At diagnosis	No
Lakatos 2003 [43]	Hungary	1977-01	212	2.23	Yes	Yes	Yes	No	At diagnosis	No
Nyhlin 1986 [51]	Sweden	1974-81	253	4.90	Yes	Yes	Yes	No	At diagnosis	No
Ruiz Ochoa 1984 [30]	Spain	1976-83	152	0.80	Yes	Yes	No	No	At diagnosis	No
Sedlack 1980 [31]	USA	1935-75	103	4.20	Yes	No	No	No	At diagnosis	No
Brandes 1983 [52]	Germany	1964-75	97	3.00	Yes	No	Yes	No	At diagnosis	No
Latour 1996 [45]	Belgium	1993-94	56	5.50	Yes	Yes	Yes	No	At diagnosis	No

**Table 2:** Characteristics of case–control studies for ulcerative colitis and Crohn’s disease studies

**Study characteristics and demographics**				**Study quality characteristics**							
**Author (year)**	**Country**	**No. of cases**	**No. of controls**	**Source of controls**	**Controls matched for:**	**Methods of diagnosis defined**	**Diagnosis based on recognized criteria**	**Urban/rural defined**	**Urban population >10,000**	**Time of exposure**	**Multiple levels of exposure**
*Ulcerative Colitis*											
Parrello 1997 [26]	Italy	509	657	Attending orthopedic and surgical clinics	Sex, age, year of diagnosis	Yes	Yes	No	No	Time of data collection	No
Feeney 2002 [17]	UK	137	137	Functional GI Disorders	Sex, age	Yes	Yes	No	No	Childhood (age 0–5)	No
Bernstein 2007 [27]	Canada	137	310	Manitoba Health Population Registry	Sex, age	No	N/A	No	No	N/A	No
Jiang 2007 [28]	China	177	177	Neighbours or colleagues	Sex, age	Yes	Yes	No	No	Childhood	No
Radon 2007 [54]	Germany	304	1481	Strabismus surgery	Age	No	N/A	No	No	Time of data collection	No
Martinez Salmeron 1994 [46]	Spain	63	63	Hospital	Sex, age	Yes	Yes	Yes	No	Time of data collection	No
Ekbom 1990 [8]	Sweden	164	328	Live births between 1924–1957 in Uppsala County	Date of birth, sex, and maternal age or parity	Yes	Yes	Yes	No	Time of birth	No
*Crohn’s Disease*											
Feeney 2002 [17]	UK	139	139	Functional GI disorders	Sex, age	Yes	Yes	No	No	Childhood (age 0–5)	No
Bernstein 2007 [27]	Canada	235	310	Manitoba Health Population Registry	Sex, age	No	N/A	No	No	N/A	No
Ponsonby 2009 [53]	Australia	278	998321	Live births from 1983–1998 in Victoria	Age	Yes	No	Yes	No	Childhood (age 0–6)	No
Malekzadeh 2009 [16]	Iran	196	207	IBS treated by an expert gastroenterologist	Age	Yes	Yes	No	No	Childhood (before 16^th^ birthday)	No
Radon 2007 [54]	Germany	444	1481	Strabismus surgery	Age	No	N/A	No	No	Time of data collection	No
Thompson 1998 [29]	England, Wales	291	1682	Same GP	Sex, age	No	N/A	No	No	N/A	No
Wurzelmann 1994 [13]	United States	322	262	Closest neighbour	Sex, age, race	No	N/A	No	No	Childhood (age 0–5)	Yes
Martinez Salmeron 1994 [46]	Spain	30	30	Hospital	Sex, age	Yes	Yes	Yes	No	Time of data collection	No
Ekbom 1990 [8]	Sweden	93	186	Live births between 1924–1957 in Uppsala County	Date of birth, sex, and maternal age or parity	Yes	Yes	Yes	No	Time of birth	No

### Urban/rural environment and IBD

**Figure 2:**
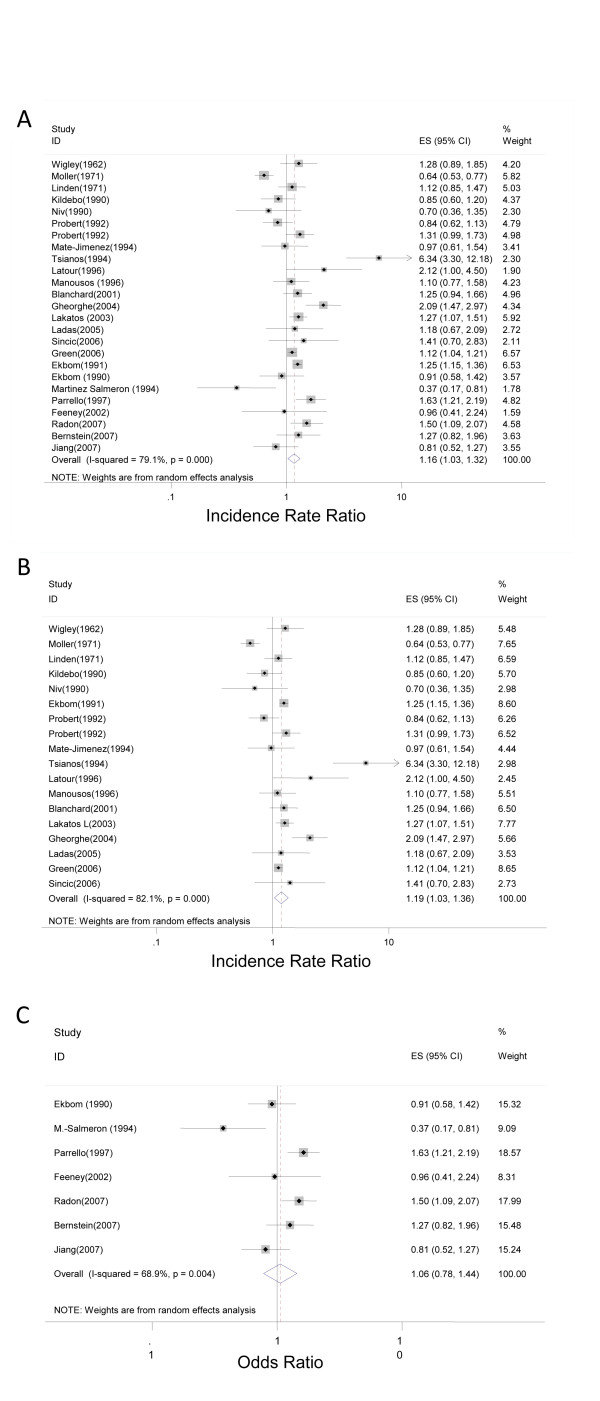
Forest plot of the summary effect estimate with 95% CI studies that explored the relationship between urban environment and ulcerative colitis for both cohort and case–control studies (A); cohort studies (B); and case–control studies (C).

**Figure 3:**
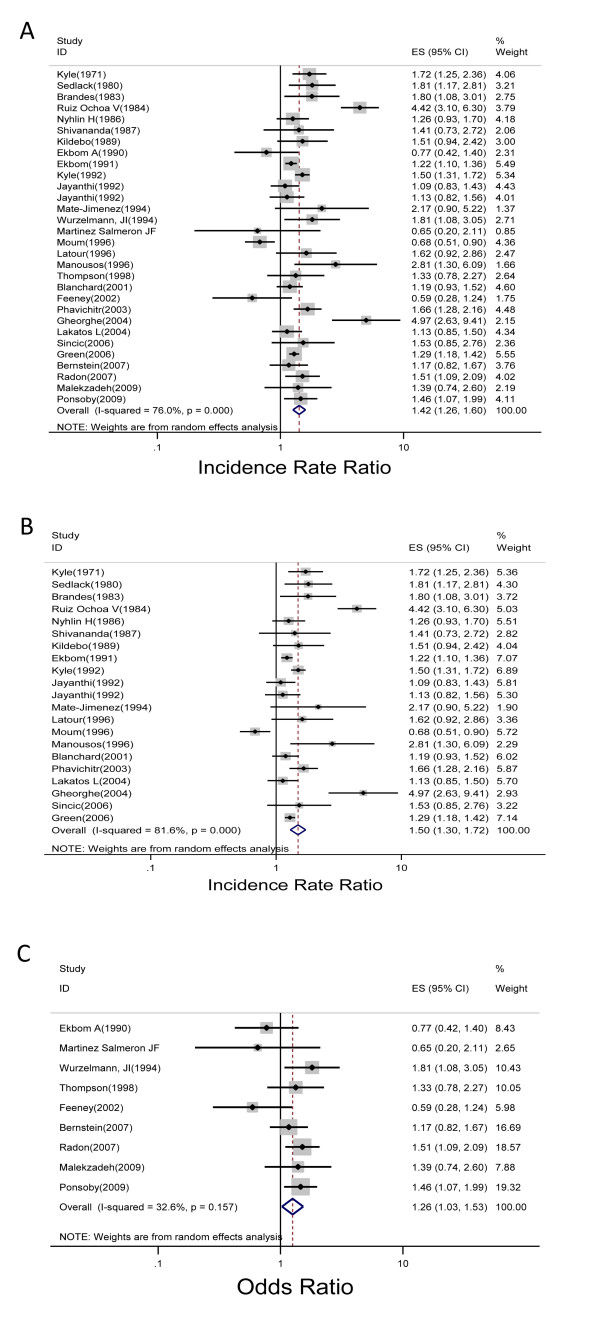
Forest plot of the summary effect estimate with 95% CI studies that explored the relationship between urban environment and Crohn’s disease for both cohort and case–control studies (A); cohort studies (B); and case–control studies (C).

The pooled crude IRR for the 25 UC studies was 1.17 (1.03, 1.32) (Figure 2A). For the 18 cohort studies, the pooled crude IRR was 1.19 (1.03, 1.36) (Figure 2B). In 13 of the studies, a positive association between UC and urban environment was found, 7 of which were statistically significant. Heterogeneity across studies was observed (Q statistic, 95.14; *P* < .001). For the UC case–control studies, the pooled OR for the association between urban environment and UC was 1.06 (0.78, 1.45) (Figure 2C). Three out of seven studies found a positive association, and two were statistically significant. Heterogeneity was observed across studies (Q statistic, 19.32; *P* = .004). The pooled IRR for the 30 CD studies was 1.42 (1.26, 1.60) (Figure 3A). For the 21 cohort studies, the pooled crude IRR was 1.50 (1.30, 1.72) (Figure 3B). In 20 of the studies, a positive association between CD and urban environment was found, 10 of which were statistically significant. Heterogeneity across cohort studies was observed (Q statistic, 108.56; *P* < .001). For the 9 CD case–control studies, the pooled OR for the association between urban environment and CD was 1.26 (1.03, 1.53) (Figure 3C). Six out of the seven studies found a positive association, but only three were statistically significant. Heterogeneity was not observed across studies (Q statistic, 11.88; *P* = .157).

### Sensitivity and stratified analyses

After excluding the UC cohort studies that did not define urban and/or rural environment the pooled IRR was 1.18 (1.09, 1.27), which did not differ from the crude pooled IRR (Table 3). The IRR lost significance 1.16 (0.9, 1.5) when we included only studies that defined urban as >10,000 people. Only cohort studies published after 1999 demonstrated an association between urban environment and UC (1.30; 95% CI: 1.10, 1.54). A stratified analysis was similarly performed for region in which the study was based, divided into European and non-European countries. The IRR for Non-European studies was statistically significant with pooled estimate of 1.13 (1.06, 1.22). Meta-regression was not statistically significant for year of publication, region of publication and European countries.

**Table 3:** Sensitivity and stratified analyses of pooled relative risk for cohort studies

	**Ulcerative colitis**						**Crohn’s disease**					
	**Stratified analysis**				**Meta-regression**		**Stratified analysis**				**Meta-regression**	
	**No. of studies**	**No. of cases**	**Pooled RR (95%CI)**	**Heterogeneity (p-value)**	**Coef.**	**P value**	**No. of studies**	**No. of cases**	**Pooled RR (95%CI)**	**Heterogeneity (p-value)**	**Coef.**	**P value**
Crude	18	6901	1.19(1.03, 1.36)	<.001			21	6514	1.50(1.30, 1.72)	<.001		
**Definition of urban/rural environment**												
Urban/rural defined	13	5906	1.18(1.09, 1.27)	.19			16	5741	1.28(1.14, 1.43)	<.001		
Urban defined as >10,000	3	299	1.16(0.90, 1.50)	.65			4	488	1.38(0.97, 1.97)	.072		
**Year of publication**												
1962-1988	3	860	0.96(0.60, 1.51)	<.001	0.16	.30	6	825	1.90(1.27, 2.85)	<.001	−0.095	.47
1989-1998	9	3459	1.23(0.95, 1.57)	<.001			9	3176	1.26(1.04, 1.53)	<.001		
1999-2009	6	2612	1.30(1.10, 1.54)	.022			6	2513	1.49(1.18, 1.87)	.001		
**Region**												
European countries	15	5036	1.21(0.99, 1.46)	<.001	0.008	.98	17	4295	1.55(1.28, 1.88)	<.001	0.77	.75
Non-European countries	3	1895	1.13(1.06, 1.22)	.61			4	2219	1.39(1.18 1.62)	.12		
**European Countries**												
Northern	6	3819	0.97(0.75, 1.27)	<.001	−0.26	.086	9	3499	1.23(1.04, 1.45)	<.001	0.23	.04
Eastern	2	723	1.59(0.98, 2.59)	.013			2	297	2.30(0.54, 9.82)	<.001		
Mediterranean	6	458	1.39(0.82, 2.36)	<.001			4	346	2.64(1.50, 4.62)	.02		
Western	1	36	2.12(1.00, 4.50)	NA			2	153	1.72(1.17, 2.51)	.79		

For the UC case–control studies, we failed to find a statistically significant association when stratifying by timing of exposure (Table 4). Meta-regression was performed for both timing of exposure and source of controls and no statistically significant associations were found.

**Table 4:** Sensitivity and stratified analyses of pooled odds ratio for case–control studies

	**Ulcerative colitis**							**Crohn’s disease**						
	**Stratified analysis**					**Meta-regression**		**Stratified analysis**					**Meta-regression**	
	**No. of studies**	**No. of cases**	**No. of controls**	**Pooled OR (95%CI)**	**Heterogeneity (p-value)**	**Coef.**	**P value**	**No. of studies**	**No. of cases**	**No. of controls**	**Pooled OR (95%CI)**	**Heterogeneity (p-value)**	**Coef.**	**P value**
Crude	7	1491	3153	1.06 (0.78, 1.45)	.004			9	2028	1002618	1.26 (1.03, 1.53)	.16		
**Time of exposure**														
Time of data collection	4	1013	2511	1.18 (0.78, 1.80)	.006	-0.32	.42	2	474	1511	1.21 (0.58, 2.50)	.18	0.060	.89
Childhood	3	478	642	0.87 (0.65, 1.17)	.91			5	1028	999115	1.18 (0.82, 1.70)	.052		
0-6 years								4	832	998908	1.12 (0.71, 1.77)	.026	0.16	.46
< 16 years								1	196	207	1.39 (0.74, 2.60)	NA		
**Source of controls**														
Population	2	314	487	1.02 (0.66, 1.58)	.16	0.035	.94	3	835	998893	1.40 (1.13, 1.73)	.37	-0.23	.32
Clinic/Hospital	5	1177	2666	1.06 (0.70, 1.60)	.003			6	1193	3725	1.09 (0.79, 1.51)	.11		

After excluding CD cohort studies that did not define urban and/or rural environment, the pooled IRR was 1.28 (1.14, 1.43), which was similar to the crude IRR (Table 3). Restricting the urban definition to >10,000 people resulted in a loss of significance with an IRR of 1.38 (0.97, 1.97). The pooled IRRs were statistically significant across all 3 categories of publication year (Table 3). A stratified analysis was similarly performed for region in which the study was based, divided into European and non-European countries. The IRRs for both European and non-European studies were statistically significant with pooled estimates of 1.55 (1.28, 1.88) and 1.39 (1.18, 1.62), respectively. In meta-regression analysis only the European countries variable was statistically significant (Table 3).

For the CD case–control studies, we failed to find a statistically significant association when stratified by timing of exposure (Table 4). Meta-regression was performed for both time of exposure and source of controls and no statistically significant associations were found. The OR for population-based controls was 1.40 (1.13, 1.73) and for hospital/clinic-based controls was 1.09 (0.79, 1.51). Meta-regression was performed for both variables and no statistically significant result was found.

### Publication bias

No publication bias was found; the Begg tests were not statistically significant for UC (z = −0.42, *P* = .675) or CD studies (z = 0.70, *P* = .487).

## Discussion

Urbanization of society is an important risk factor for the development of IBD. This meta-analysis suggests that living in urban environments may increase the risk of developing CD and UC. Though, the strengths of association varied among the 40 studies due to heterogeneity between studies. The association between CD and urban environment persisted across a number of stratified analyses that explored clinical and study quality factors. In contrast, the association between UC and urban environment was weaker and less consistent upon sensitivity analyses. Publication bias was not observed suggesting that the association were likely not an artifact of unpublished studies.

Several theories may explain the increased incidence of IBD in urban societies. The Hygiene Hypothesis proposes that the lack of early childhood exposure to enteric pathogens with improved sanitation in urban cities increases the incidence of IBD [5,55]. The lack of exposure to enteric pathogens may lead to a greater susceptibility to develop an inappropriate immunologic response upon exposure to new antigens (e.g. gastrointestinal infection) later in life [56]. Other environmental risk factors of IBD that are more predominant in urban societies include smoking, lack of helminths exposure, and antibiotic use [57-59]. Additionally, a recent study demonstrated that air pollution in urban cities was associated with IBD in children [60]. Urban occupations such as driving and manufacturing have also been reported as risk factors for IBD [61]. While a cohesive hypothesis that explains the environmental determinants of IBD has not been proven, this meta-analysis supports further research exploring the environmental risk factors of urbanization and IBD.

Heterogeneity was observed in both the CD and UC cohort studies, whereas statistically significant heterogeneity was not observed for the UC and CD case–control studies. This inconsistency was likely due to the large overall study population in the cohort studies, leading to an overpowered heterogeneity statistical test. In contrast, the case–control studies were small in total study population. Alternatively, differences in heterogeneity between studies may be explained by the difference in the study design and by the intrinsic biases associated with cohort and case–control studies that explore environmental risk factors of IBD.

When studies were stratified by region the association between UC and urban environment persisted only in non-European studies, whereas the association between CD and urban environment remained significant in most regions. Regional differences may be due to the fact that studies originated from countries that differed by ethnicity, prevalence of IBD, and rates of IBD susceptibility genes [62,63]. Alternatively, the small number of studies in each region made appropriate and meaningful inferences challenging. Furthermore, data was lacking for low prevalent regions (i.e. developing world) and thus we could not explore the urban/rural effect in these regions.

We explored whether the year of publication contributed to the heterogeneity observed between studies. For UC, only publications after 1999 were significant after pooling. This time stratified finding may be due to study design differences in more recent publications or the changing pattern of UC diagnosis. In contrast, the urban/rural relationship persisted across all time strata for CD, which suggested a greater strength of association for CD over UC.

The definition of an urban environment was a source of heterogeneity across studies. The studies that clearly defined this exposure included population estimates of the urban and/or rural areas; however, the inconsistency or lack of definitions made study comparisons challenging. We a priori selected the definition of urban as more than 10,000 people because this definition is consistent with census of populations in many Countries (e.g. United Kingdom [37] and Canada [38]). When considering only studies that defined urban as >10,000, the association was no longer significant, which was likely due to the small number of studies with this definition (n = 2 for UC and n = 4 for CD). However, this finding may also reflect that the risk associated with urban society may be driven by greater population size (e.g. living in a metropolis) and/or by population density. Ideally, we would have investigated the potential for a dose response effect to explore whether the risk of IBD increased with increasing population sizes. However, few papers stratified their results by multiple levels of exposure; for example, Tsianos [24] and Ladas [34] stratified their analysis into urban, semi-rural, and rural. Future studies of the urban–rural association should explore the importance of a dose response effect, a threshold value of population size, and population density.

Heterogeneity between studies may have occurred due to the biases associated with cohort and case–control studies. For the case–control studies, a selection bias may have influenced the association if cases were selected by a different mechanism than controls. The controls were grouped into population- and hospital/clinic-based selection categories. For CD, only population-based controls demonstrated a significant association with urban environment; in contrast, the urban–rural association was insignificant in both control populations for UC. Additionally, inconsistency in timing of exposure (e.g. defining urban–rural status at time of diagnosis versus childhood) may have contributed to the heterogeneity between case–control studies. People in rural settings may have less access to health care leading to under diagnosis of IBD. Although the majority of the case–control studies were matched by age and gender, other potential confounders including socioeconomic status and smoking were often not considered. Finally, studies that used administrative databases to identify IBD patients likely introduced misclassification errors. Interpreting the results of meta-analysis should be cautious because pooling data does not address the intrinsic biases of observational studies.

Limitations in our systematic review should be considered. First, the number of studies included in the stratified analyses was small and may have been underpowered. Second, the quality of studies was not always optimal as was shown with the inconsistent definitions of urban/rural and time of exposure. Third, the included studies used population estimates as indicators for urban environment; however, other factors that contribute to an urban setting, such as socioeconomic characteristics, were not considered. Finally, due to the nature of observational studies, a temporal relationship could not be determined. Thus, urban environment cannot be conclusively established as a causal factor in IBD.

## Conclusions

In spite of differences in study design and population characteristics, the meta-analysis demonstrated that living in an urban society was positively associated with the development of IBD; though, the consistency and strength of the association was greatest for CD. Additionally, the meta-analysis identified important study limitations and thus, future studies should be properly designed using a standard definition of urban/rural. Furthermore, additional studies are need to evaluate whether the following factors affect the risk of developing IBD: increasing population size (i.e. a dose response effect); a threshold value of population size; the number of people living per unit area (i.e. population density); and the duration and timing of exposure. Finally, the meta-analysis highlights that researchers should continue to explore for environmental differences between urban and rural societies.

## Abbreviations

CD, Crohn’s disease; UC, ulcerative colitis; IRR, incidence rate ratio; OR, odds ratio; IBD, inflammatory bowel disease; MESH, Medical Subject Headings; CI, confidence interval.

## Competing interests

None.

## Author contributions

*G K.* Dr. Kaplan participated in conceiving the study idea, developing the study design, interpreting results, and writing of the manuscript. Dr. Kaplan confirms that he has had full access to all the data in the study and had final responsibility for the decision to submit for publication. Dr. Kaplan has seen and approved the final version of the manuscript and has no relevant conflicts of interest. *I S S.* Dr. Soon participated in conceiving the study and design, acquisition of data, analysis, interpretation of data, drafting manuscript, and statistical analysis*.* Dr. Soon has seen and approved the final version of the manuscript and has no relevant conflicts of interest. *N M.* Ms Molodecky participated in conceiving the study concept and design, acquisition of data, analysis and interpretation of data, drafting manuscript, and statistical analysis. Ms Molodecky has seen and approved the final version of the manuscript and has no conflicts of interest. *D R.* Dr. Rabi participated in conceiving the study study concept and design, analysis and interpretation of data, and critical revision of the manuscript for important intellectual content. Dr. Rabi has seen and approved the final version of the manuscript. Dr. Rabi has no conflicts of interest. *W G.* Dr. Ghali participated in analysis and interpretation of data, and critical revision of the manuscript for important intellectual content. Dr. Ghali has seen and approved the final version of the manuscriptand has no conflicts of interest. *H B.* Dr. Barkema participated in analysis and interpretation of data, and critical revision of the manuscript for important intellectual content. Dr. Barkema has seen and approved the final version of the manuscript, and has no conflicts of interest.

## Source of funding

This research is supported by The Alberta IBD Consortium, which is funded by an AHFMR Interdisciplinary Team Grant. AHFMR is now Alberta Innovates - Health Solutions.

## Pre-publication history

The pre-publication history for this paper can be accessed here:

http://www.biomedcentral.com/1471-230X/12/51/prepub
